# Associations with food allergy‐related psychological distress in a global sample of adults, children and caregivers

**DOI:** 10.1002/clt2.70071

**Published:** 2025-06-06

**Authors:** S. E. M. Purser, C. J. Jones, J. L. P. Protudjer, L. J. Herbert, C. Screti, C. Roleston, E. Mattacola, H. A. Brough, C. Warren, L. Polloni, A. F. Santos, R. Gupta, M. J. Marchisotto, R. C. Knibb

**Affiliations:** ^1^ School of Psychology Aston University Birmingham UK; ^2^ School of Psychology Faculty of Health and Medical Sciences University of Surrey Guildford UK; ^3^ Department of Pediatrics and Child Health University of Manitoba Winnipeg Manitoba Canada; ^4^ Children's Hospital Research Institute of Manitoba Winnipeg Manitoba Canada; ^5^ Institute of Environmental Medicine Karolinska Institutet Stockholm Sweden; ^6^ Children's National Hospital Washington District of Columbia USA; ^7^ George Washington University School of Medicine Washington District of Columbia USA; ^8^ Aston Institute for Health and Neurodevelopment Aston University Birmingham UK; ^9^ Nuffield Department of Primary Care Health Sciences University of Oxford Oxford UK; ^10^ Children's Allergy Service Evelina Children's Hospital Guy's and St. Thomas's NHS Foundation Trust London UK; ^11^ Department Women and Children's Health (Pediatric Allergy) School of Life Course Sciences Faculty of Life Sciences and Medicine King's College London London UK; ^12^ Peter Gorer Department of Immunobiology School of Immunology and Microbial Sciences King's College London London UK; ^13^ Center for Food Allergy and Asthma Research Northwestern University Feinberg School of Medicine Chicago Illinois USA; ^14^ Department of Women's and Children's Health Food Allergy Referral Centre Veneto Region Padua University Hospital Padua Italy; ^15^ Unit of Psychology Padua University Hospital Padua Italy; ^16^ Ann & Robert H. Lurie Children's Hospital of Chicago Chicago Illinois USA; ^17^ Institute for Public Health and Medicine Northwestern Feinberg School of Medicine Chicago Illinois USA; ^18^ MJM Advisory New York New York USA

**Keywords:** adults, caregivers, children, food allergy, psychological distress

## Abstract

**Objective:**

Food allergy (FA) impacts health‐related quality of life and mental health. Understanding what variables are associated with psychological distress can help healthcare providers direct patients to appropriate support. As part of the study, Global Access to Psychological Services (GAPS) for FA and associations with FA‐related psychological distress were explored in adults with FA and caregivers of children with FA.

**Methods:**

Participants completed online surveys in seven languages. Participants reported the types of FA‐related distress they or their child experienced, along with demographic and FA‐related information. Associations with distress were analysed using regression models.

**Results:**

*N* *=* 1329 adults with FA and *N* = 1373 caregivers of children with FA from 27 countries participated. Of the 21 different types of distress selected, anxiety about an allergic reaction was the most common (62.5% adults; 72.6% caregivers). Females reported significantly more types of distress than males (*p* < 0.001). There were significant differences between countries (all *p* < 0.05‐0.001); participants in Australia, Brazil, Canada, and the United Kingdom consistently reported more types of distress than European countries or the United States. In regression models, country of residence, number of FAs, and symptoms were significantly associated with distress. Additional associations included adrenaline autoinjector (AAI) prescription, being female, anaphylaxis and comorbidities in adults; in caregivers having a younger child, longer time elapsed since FA diagnosis, being female, AAI prescription and anaphylaxis; and in children being older and living longer with FA.

**Conclusions:**

FA‐related distress is experienced differently across countries. Understanding associations with types of distress can help direct healthcare services and psychological support to where it is needed most.

## 1 INTRODUCTION

Food allergy (FA) has a significant and negative effect on the health‐related quality of life and mental health of affected adults, children, and caregivers.^1^, ^2^, ^3^ Our recent Global Access to Psychological Services (GAPS) for Food Allergy survey identified substantial and heterogeneous dimensions of FA‐related psychological distress across North American, South American, European, and Australasian FA adults, children and caregivers.^4^

Research suggests that certain sociodemographic and clinical characteristics of patients with FA may affect their FA‐related quality of life (FAQoL). These include being allergic to multiple foods,^5^, ^6^ perceived disease severity,^7^ being female,^8^, ^9^, ^10^ allergen type,^6^, ^7^ and reaction symptomatology.^7^ In adults, history of anaphylaxis and adrenaline autoinjector (AAI) prescription negatively impact QoL.^6^, ^9^ Children and caregivers can develop acute stress disorder after anaphylaxis^11^ and anaphylaxis may impact the caregiver years after, even when the adolescent cannot remember the reaction and reported little impact on their day‐to‐day life.^12^ In children and caregivers, anxiety has been reported to be unrelated to previous reactions to food but AAI prescription was associated with reduced anxiety.^13^ However, key confounders such as FA severity and trait anxiety were not adjusted for, which could have influenced clinician decisions regarding AAI prescription. There is some evidence that AAI can be a source of reassurance with one study finding that for 81.8% of 13–17 year olds, carrying AAI provided reassurance whilst parents reported only 45.0% of 0–12 year olds felt the same, suggesting that age may also be a factor.^14^ The relationship between AAI prescription and the experience of anaphylaxis is complex as they have each been demonstrated to exert an independent negative effect on FAQoL.^15^ This suggests that they may do so through different mechanisms, such as the perception of the severity of their allergy.

Perceived FA severity rather than experience of anaphylaxis and AAI prescription has been found to be a significant predictor of FAQoL in children and adults.^7^ In a European study, data from 404 adults from the Netherlands, Greece, Italy, France, Iceland, Spain and Poland, and 244 children from the Netherlands, Spain, Ireland and Poland was analysed. In the adult sample, after adjusting for significant predictors (perceived disease severity, type of most severe symptom, type of food allergy and gender), there were no overall differences in FAQoL between countries. However, significant differences between countries in the emotional impact of FA and FA‐related health domains were identified. In the child sample, country of origin along with allergen type and perceived disease severity were independent predictors of FAQoL.^7^ A further study found that despite a similar number of allergens and types of symptoms reported, participants from the United States of America (USA) had significantly lower FAQoL compared to Dutch participants.^16^ However, significantly more participants from the USA had AAI and this was not adjusted for in the analysis.

The above research has identified multifaceted characteristics associated with patient and caregiver FAQoL. However, many of the cited studies are over a decade old, which constrains their applicability to current FA management contexts. For example, changes in AAI prescription guidelines^17^ and the shift in FA management from passive avoidance to active interventions to reduce reaction severity, prevent accidental exposure and improve FAQoL.^18^ Sample sizes have often been limited in number and in terms of diversity, restricting the generalisability of findings. Furthermore, many failed to account for potential confounders. The GAPS study offers the opportunity to analyse these variables collectively instead of individually to gain a better understanding of the dynamics in a large and geographically diverse sample. Previous research demonstrating inconsistent findings regarding FAQoL across countries suggests that further research is required to determine if participant characteristics explain the reported differences in psychological distress. Moreover, given the high rates of distress and the global difficulties in accessing psychological support for FA,^19^, ^20^ providers who seek to direct mental health care to their FA patients and families would benefit from a greater understanding of what variables are associated with different types and amounts of distress among their patients. Based on this, we explored the data collected as part of the GAPS study,^4^ to explore differences in FA‐specific types of distress experienced by adults, caregivers, and children based on clinical and demographic characteristics.

## 2 METHODS

### 2.1 Design

The study was based on data from a cross‐sectional online survey. Full details of the survey development and recruitment process have been published elsewhere.^4^ Ethical approval was granted by the Aston University's School of Life and Health Sciences Research Ethics Committee (REC ID #1621). All participants provided informed consent.

### 2.2 Measures

As no measure exists to examine FA psychological distress, an existing measure developed by GAPS team members^19^ was adapted to assess types of FA‐related psychological distress by the international multilingual research team with globally recognised expertise in FA, including clinical/translational science, population health/survey research, and patient advocacy. To assess FA‐related psychological distress, adults and caregivers were asked if they had experienced psychological distress related to FA. Those who said yes were able to select up to 21 types of distress they had experienced. Caregivers were able to select up to 13 types of distress their child had experienced. Examples of areas of distress included anxiety about living with food allergy, anxiety about having an allergic reaction, bullying and sadness about food allergy. The full list can be seen in the tables in the online supplement. The surveys were developed in English, and subsequently translated into French (European and Canadian variants), German, Italian, Portuguese (European and Brazilian variants), and Spanish. Translations were carried out by a translation company and successfully back‐translated by members of the study team fluent in that language.

### 2.3 Participants and procedures

Participants were adults (aged 18+ years) and caregivers (aged 18+ years) of children (aged 0–17 years) who reported a clinical diagnosis of FA by a healthcare professional. Participants were recruited via a link distributed by FA patient organisations, social media advertisements, and an online survey panel supplied by Qualtrics. The link included a participant information sheet, consent form, and the survey.

### 2.4 Data analysis

This exploratory analysis was performed in SPSS v29 (Chicago, USA). Associations with distress and correlational analysis are reported across the whole sample split by adults, caregivers and children. Independent samples *t*‐tests examined sex differences in reporting the types of distress. A one‐way analysis of variance was run to examine the effect of education on the number of types of distress experienced. Due to unequal group sizes and a significant Levene's statistic (*p* < 0.001), Welch's statistic was reported. Post hoc comparisons used the Games–Howell procedure. Associations between the types of distress experienced and FA‐related symptomatology, comorbidities, allergens, time living with FA, and the child's age were examined using Spearman Rho (where data did not meet assumptions for parametric tests) and Pearson's correlations. Chi‐square tests of independence were run for each type of distress reported by adults, caregivers and caregivers on behalf of children to compare differences in frequency of reporting individual types of distress between countries.

To explore which variables could explain the types of adult, caregiver, and child distress, multiple linear regression models were fitted. Dummy coding was used for categorical variables with more than two levels. Variables significant in the simple linear regressions were entered into the corresponding models. Clinical and demographic characteristics were entered into block one, and countries into block two.

## 3 RESULTS

### 3.1 Sample characteristics

In total, 1329 adults and 1373 caregivers completed the survey and provided data for all key analytic variables (See Table 1 for summary information and Tables A and B in the supplement for full details). Most participants identified as White females (60.5% adults, 60.0% caregivers), with a mean age of 38.1 ± 13.4 years (adults) and 37.6 ± 8.8 years (caregivers). Over half of adults (52.8%) and caregivers (57.3%) were educated to university level and in full time employment.

**TABLE 1:** Summary participant characteristics and adult and child FA characteristics (adults *N* = 1329, Caregivers and children *N* = 1373).

Participant characteristics	Adults *N* (%)	Caregivers *N* (%)
Country of residence		
Australia	120 (9.0%)	118 (8.6%)
Brazil	90 (6.8%)	188 (13.7%)
Canada	208 (15.7%)	186 (13.5%)
France	111 (8.4%)	120 (8.7%)
Germany	115 (8.7%)	110 (8.0%)
Italy	126 (9.5%)	18 (1.3%)
Portugal	130 (9.8%)	171 (12.5%)
Spain	145 (10.9%)	151 (11%)
United Kingdom	116 (8.7%)	118 (8.6%)
United States of America	122 (9.2%)	121 (8.8%)
Other	46 (3.5%)	72 (5.3%)
Age (years) (mean)	38.1 (SD = 13.4)	37.6 (SD = 8.8)
Sex (% female)	931 (70.2%)	1031 (75.1%)
Ethnicity (% white)	1135 (85.4%)	1118 (81.4%)
Highest level of education		
No qualifications	4 (0.3%)	26 (1.9%)
School level	212 (16.1%)	140 (10.2%)
College level	359 (27.2%)	384 (28.0%)
University undergraduate level	382 (29.0%)	409 (29.8%)
University postgraduate level	259 (19.7%)	304 (22.1%)
PhD or MD	54 (4.1%)	74 (5.4%)
Other	48 (3.6%)	32 (2.3%)
Employed (% employed)	912 (70.3%)	1062 (78.2%)
Marital status (% married)	558 (42.7%)	934 (68.0%)

FA characteristics of adults and children	Adults *N* (%)	Children *N* (%)
Age (years) (mean)	38.1 (SD = 13.4)	8.1 (SD = 5.3)
Age of food allergy diagnosis (years) (mean)	18.0 (SD = 13.6)	3.1 (SD = 3.7)
Time living with food allergy (years) (mean)	20.0 (SD = 13.5)	5.1 (SD = 4.2)
Allergens^a^		
Peanut	480 (36.1%)	571 (41.6%)
≥1 tree nut	411 (30.9%)	463 (33.7%)
Cow's milk	357 (26.9%)	618 (45.0%)
Egg	171 (12.9%)	472 (34.4%)
Seafood	344 (25.9%)	237 (17.3%)
≥1 other	743 (55.9%)	628 (45.7%)
≥1 FA‐related comorbid condition	1097 (82.5%)	1047 (76.3%)
Adrenaline auto‐injector prescription	652 (49.1%)	787 (57.3%)
History of anaphylaxis	666 (50.1%)	691 (50.3%)

^a^ The items listed under these headings are not mutually exclusive, that is, participants may fall into multiple categories.

Half (50.1% adults and 50.3% children) had a history of anaphylaxis and the majority had been diagnosed with at least one comorbid allergic disease. Children had a mean age of 8.1 ± 5.3 years and had been living with FA for 5.1 ± 4.2 years. The most common paediatric food allergen was cow's milk (45.0%), followed by peanut (41.6%), and egg (34.4%). Adults had been living with FA for 20.0 ± 13.5 years. The most reported food allergens were peanuts (36.1%) and cow's milk (26.9%).

### 3.2 Associations between demographic variables and reported distress

In adults, males (*n* = 385) reported significantly fewer types of FA‐related psychological distress (*M* = 2.46, SD = 3.54) compared to females (*n* = 931, *M* = 6.20, SD = 5.47), a mean difference of −3.74 (95% CI, −4.24 to −3.24) *t*(1082.80) = 14.70, *p* < 0.001. This represents a large effect size, Cohen's *d* = −0.81 (95% CI, −0.93 to −0.69). Male caregivers (*n* = 333) also reported significantly fewer types of FA‐related psychological distress (*M* = 2.73, SD = 4.07) compared to females (*n* = 1031, *M* = 8.55, SD = 6.01), a mean difference of −5.82 (95% CI, −6.39 to −5.25), *t*(831.52) = ‐19.97, *p* < 0.001. This represents a large effect size, Cohen's *d* = −1.13 (95% CI, −1.26 to −1.00). A child's biological sex (male/female) did not affect the number of types of distress a child reportedly experienced, *t*(1357) = 0.03, *p* = 0.588.

For adults, caregivers, and children, there were significant positive associations between experiencing more types of distress and being allergic to more foods, being diagnosed with more comorbid conditions, and experiencing more FA symptoms (all *p* < 0.001). Children and caregivers were likely to report more types of distress the longer they had been living with FA (all *p* < 0.001) (See Table 2).

**TABLE 2:** Associations with the sum of the types of distress in adults, caregivers and children.

	Adult sum of types of distress	Caregiver sum of types of distress ***ρ***	Child sum of types of distress ***ρ***
Time living with FA (years)	−0.04	0.2*	0.46*
Sum of allergens^a^ *ρ*	0.53*	0.44*	0.26*
Sum of comorbid conditions^b^	0.44*	0.27*	0.37*
Sum of all symptoms^c^	0.60*	0.51*	0.46*
Child age (years)	–	−0.13*	0.38*

*Note*: *ρ* denotes Spearman correlations.

^a^ Sum of allergens was measured on a continuous scale. To calculate the sum of allergens, a total of allergens selected by each participant was calculated. Those who indicated ‘other’ food allergens and then listed them were counted and added to the total. Those who wrote ‘most nuts’ but had not selected any were excluded from all analyses that used this variable.

^b^ Sum of 9 co‐morbidities; see table B in the supplementary file for full list.

^c^ Sum of 34 symptoms that participants reported as a reaction to food; see supplementary file for full list.

*p < 0.001 (two‐tailed).

There was a significant effect of education level on the sum of types of distress experienced by adults as determined by Welch's *F*(6, 43.02) = 3.86, *p* = 0.004. Adults with a PhD or MD reported significantly fewer types of distress than adults with a university undergraduate education (*p* < 0.001), a university postgraduate education, or other qualification (*p* = 0.11). There was also a significant effect of caregiver education level on the sum of the types of distress experienced as determined by Welch's *F*(6175.01) = 5.81, *p* < 0.001. Caregivers with less educational attainment reported significantly more types of distress than other groups (*p* < 0.001 to 0.040). Caregiver education on child distress had no effect (Welch's *F*(6173.10) = 1.79, *p* = 0.105) (See Table C in supplement).

### 3.3 Associations between country of residence and types of distress

There were significant differences (*p* < 0.001) between countries for all types of distress across adults and caregivers (See Table 3). All were significant at the *p* < 0.001 level apart from adult ‘needle phobia or other medical procedure’, for which *p* = 0.033. In adults and caregivers, higher percentages of participants from Australia, Brazil, Canada and the UK consistently reported experiencing most types of distress. Lower percentages of participants in mainland Europe and the USA reported experiencing each type of distress. The exception was that adults in Brazil did not report as much distress around AAIs and oral food challenges. UK caregivers' concerns about cost were low and comparable to countries in mainland Europe; comparatively, cost was a significant concern for caregivers in Brazil and Australia.

**TABLE 3:** Five most common types of distress reported by adults, children and caregivers compared across countries.

		*N*	Anxiety about an allergic reaction	Anxiety about living with food allergy	Sadness about the impact of food allergy on life	Worry about a potentially fatal reaction	Worry about not being able to take part in social activities
Australia	Adult	120	77 (64.2%)	70 (58.3%)	40 (33.3%)	49 (40.8%)	48 (40%)
	Caregiver	118	107 (90.7%)	92 (78%)	98 (83.1%)	97 (82.2%)	76 (64.4%)
	Child		62 (52.5%)	50 (42.4%)	62 (52.5%)	38 (32.2%)	47 (39.8%)
Brazil	Adult	90	68 (75.6%)	64 (71.1%)	63 (70%)	41 (45.6%)	51 (56.7%)
	Caregiver	188	134 (71.3%)	151 (80.3%)	131 (69.7%)	100 (53.2%)	103 (54.8%)
	Child		47 (25%)	51 (27.1%)	62 (33%)	31 (16.5%)	45 (23.9%)
Canada	Adult	208	141 (67.8%)	123 (59.1%)	100 (48.1%)	103 (49.5%)	90 (43.3%)
	Caregiver	186	122 (65.6%)	104 (55.9%)	92 (49.5%)	38 (20.4%)	69 (34.9%)
	Child		80 (43%)	68 (36.6%)	71 (38.2%)	50 (26.9%)	55 (29.6%)
France	Adult	111	24 (21.6%)	35 (31.5%)	13 (11.7%)	5 (4.5%)	7 (6.3%)
	Caregiver	120	31 (25.8%)	36 (30%)	10 (8.3%)	9 (7.5%)	11 (9.2%)
	Child		19 (15.8%)	37 (30.8%)	12 (10%)	6 (5%)	8 (6.7%)
Germany	Adult	115	33 (28.7%)	29 (25.2%)	21 (18.3%)	5 (4.3%)	5 (4.3%)
	Caregiver	110	33 (30%)	34 (30.9%)	14 (12.7%)	12 (10.9%)	9 (8.2%)
	Child		26 (23.6%)	23 (20.9%)	13 (11.8%)	10 (9.1%)	11 (10%)
Italy	Adult	126	50 (39.7%)	54 (42.9%)	29 (23%)	26 (20.6%)	21 (16.7%)
Portugal	Adult	130	42 (32.3%)	31 (23.8%)	26 (20%)	15 (11.5%)	11 (8.5%)
	Caregiver	171	89 (52%)	76 (44.4%)	56 (32.7%)	65 (38%)	37 (21.6%)
	Child		39 (22.8%)	41 (24%)	45 (26.3%)	26 (15.2%)	29 (17%)
Spain	Adult	145	53 (36.6%)	51 (35.2%)	36 (24.8%)	29 (20%)	27 (18.6%)
	Caregiver	151	50 (33.1%)	40 (26.5%)	31 (20.5%)	34 (22.5%)	26 (17.2%)
	Child		16 (10.6%)	26 (17.2%)	24 (15.9%)	15 (9.9%)	15 (9.9%)
UK	Adult	116	91 (78.4%)	82 (70.7%)	64 (55.2%)	79 (68.1%)	65 (56%)
	Caregiver	118	76 (64.4%)	76 (64.4%)	60 (50.8%)	65 (55.1%)	52 (44.1%)
	Child		54 (45.8%)	51 (43.2%)	47 (39.8%)	35 (29.7%)	36 (30.5%)
USA	Adult	122	46 (37.7%)	47 (38.5%)	23 (18.9%)	19 (15.6%)	13 (10.7%)
	Caregiver	121	69 (57%)	71 (58.7%)	46 (38%)	44 (36.4%)	35 (28.9%)
	Child		42 (34.7%)	52 (43%)	40 (33.1%)	25 (20.7%)	24 (19.8%)

*Note*: Cells with an orange fill shows where the adjusted residual was ≤2, which shows that significantly more participants reported that type of distress than expected. Cells with a green fill shows cells where the adjusted residual was ≤−2 which shows that significantly fewer participants than expected reported distress. Due to insufficient participant numbers, Italy was excluded as a comparative group for caregivers and children.

For types of distress caregivers reported on behalf of their children (Table 3), there were significant differences between countries for all types of distress (*p* < 0.001) apart from children's worry about managing health (*p* = 0.056). Children from Australia, Canada, the UK and the USA appeared to experience more types of distress than those from mainland Europe and Brazil. In addition, higher percentages of caregivers appeared to report experiencing individual types of distress themselves than they did for their children. For example, across countries 30.0%–90.7% of caregivers experienced anxiety about their child having an allergic reaction compared to 10.6%–52.5% of caregivers stating their child experienced anxiety about an allergic reaction.

Table 3 shows the most commonly reported types of distress applied to adults, caregivers and children. For a full breakdown of every type of distress across country by adult, caregiver and child separately, see Tables D and E in the supplementary file.

### 3.4 Associations with types of FA‐related psychological distress

#### 3.4.1 Adults

The regression model for adults accounted for 51% variance in the number of types of FA‐related psychological distress reported by adults. The strongest variable was a greater number of symptoms (*β* = 0.27, 95% CI 0.17 to 0.26). Other variables that significantly contributed were AAI prescription, a greater number of allergens, being female, having experienced anaphylaxis or being unsure whether anaphylaxis was experienced, a greater number of comorbidities, and country of residence (Table 4).

**TABLE 4:** Hierarchical regression model of variables associated with the types of FA related psychological distress in adults (*N* = 1257).

Predictor	*N*	Model 1	Model 2
		Standardised *β* | Unstandardised *β* (95% CI)	
Sum of allergens		0.18 | 0.21 (0.15 to 0.27)***	0.15 | 0.18 (0.13 to 0.24)***
Sum of comorbidities		0.08 | 0.28 (0.10 to 0.46)**	0.07 | 0.24 (0.07 to 0.42)**
Sum of symptoms		0.32 | 0.26 (0.21 to 0.31)***	0.27 | 0.22 (0.17 to 0.26)***
Sex			
Male (reference)	379	–	–
Female	878	0.14 | 1.60 (1.09 to 2.09)***	0.10 | 1.11 (0.62 to 1.61)***
Prescribed AAI			
No (reference)	583	–	–
Yes	611	0.19 | 2.01 (1.43 to 2.59)***	0.18 | 1.85 (1.25 to 2.44)***
Unsure	63	−0.02 | −0.53 (−1.57 to 0.52)	−0.01 | −0.28 (−1.30 to 0.73)
Experienced anaphylaxis			
No (reference)	507	–	–
Yes	620	0.09 | 0.97 (0.36 to 1.57)**	0.07 | 0.75 (0.17 to 1.34)*
Unsure	130	0.07| 1.16 (0.37 to 1.95)**	0.05 | 0.93 (0.17 to 1.70)*
Treated with adrenaline			
No	623		
Yes	509	−0.05 | −0.53 (−1.11 to 0.05)	−0.02 | −0.18 (−0.75 to 0.38)
Unsure	125	−0.02 | −0.42 (−1.21 to 0.38)	−0.01 | −0.17 (−0.94 to 0.60)
Country			
Canada (reference)	202	–	–
Australia	117	–	−0.01 | −0.09 (−0.95 to 0.77)
Brazil	90	–	0.08 | 1.68 (0.71 to 2.66)***
France	109	–	−0.11 | −1.95 (−2.88 to −1.03)***
Germany	115	–	−0.10 | −1.86 (−2.77 to −0.95)***
Italy	126	–	−0.06 | −1.01 (−1.88 to −0.15)*
Portugal	123	–	−0.12 | −2.06 (−2.94 to −1.17)***
Spain	145	–	−0.13 | −2.08 (−2.89 to −1.26)***
UK	114	–	0.03 | 0.48 (−0.39 to 1.35)
USA	116	–	−0.12 | −2.12 (−2.99 to −1.25)***
*R* ^2^		0.464	0.506
Adjusted *R* ^2^		0.460	0.490
*R* ^2^ change		0.464	0.042
*F* statistic		107.81 (*p* < 0.001)	66.58 (*p* < 0.001)

*p < 0.050, **p < 0.010, ***p < 0.001.

#### 3.4.2 Caregivers

The regression model for caregivers accounted for 54% variance in the number of types of FA‐related psychological distress reported by caregivers. The strongest variable was a greater number of symptoms (*β* = 0.25, 95% CI 0.23 to 0.36). Other variables that significantly contributed were the child being younger, living longer with their child's FA, a greater number of allergens, biologically female caregivers, AAI prescription, history of anaphylaxis, and country of residence (Table 5).

**TABLE 5:** Hierarchical regression model of variables associated with the types of FA related psychological distress in caregivers (*n* = 1210).

Predictor	*N*	Model 1	Model 2
		Standardised | Unstandardised *β* (95% CI)	
Sum of allergens		0.17 | 0.27 (0.19 to 0.35)***	0.15 | 0.25 (0.17 to 0.32)***
Time living with food allergy		0.26 | 0.38 (0.28 to 0.48)***	0.18 | 0.26 (0.16 to 0.36)***
Sum of comorbidities		−0.07 | −0.35 (−0.6 to −0.09)**	−0.01 | −0.05 (−0.30 to 0.2)
Sum of symptoms		0.33 | 0.39 (0.33 to 0.45)***	0.25 | 0.29 (0.23 to 0.36)***
Child's age		−0.31 | −0.38 (−0.46 to −0.29)***	−0.20 | −0.24 (−0.33 to −0.16)***
Caregiver sex			
Male (reference)	303	–	–
Female	907	0.20 | 2.88 (2.23 to 3.53)***	0.14 | 1.96 (1.33 to 2.59)***
Child prescribed AAI			
No (reference)	452	–	–
Yes	711	0.13 | 1.66 (0.98 to 2.33)***	0.14 | 1.70 (1.03 to 2.37)***
Unsure	47	−0.03 | −1.08 (−2.47 to 0.31)	−0.01 | −0.42 (−1.75 to 0.92)
Child experienced anaphylaxis			
No (reference)	512	–	–
Yes	616	0.12 | 1.45 (0.75 to 2.16)***	0.11 | 1.33 (0.66 to 1.99)***
Unsure	82	−0.01 | −0.26 (−1.34 to 0.81)	0.01 | 0.28 (−0.74 to 1.30)
Child treated with adrenaline			
No (reference)	678	–	–
Yes	484	−0.08 | −0.97 (−1.64 to −0.30)**	−0.05 | −0.59 (−1.23 to 0.05)
Unsure	48	−0.04 | −1.35 (−2.72 to 0.14)	−0.03 | −1.04 (−2.34 to 0.26)
Country			
Canada (reference)	180	–	–
Australia	117	–	0.08 | 1.75 (0.74 to 2.77)***
Brazil	181	–	0.14 | 2.38 (1.42 to 3.35)***
France	108	–	−0.14 | −3.09 (−4.14 to −2.03)***
Germany	99	–	−0.10 | −2.17 (−3.28 to −1.06)***
Portugal	161	–	−0.06 | −1.17 (−2.09 to −0.25)*
Spain	142	–	−0.15 | −2.78 (−3.74 to −1.83)***
UK	110	–	−0.03 | −0.73 (−1.74 to 0.29)
USA	112	–	−0.05 | −1.00 (−2.02 to 0.01)
*R* ^2^		0.479	0.540
Adjusted *R* ^2^		0.473	0.532
*R* ^2^ change		0.479	0.061
*F* statistic		91.53 (*p* < 0.001)	69.74 (*p* < 0.001)

*Note*: Due to insufficient participant numbers, Italy was excluded as a comparative group.

*p < 0.050, **p < 0.010, ***p < 0.001.

#### 3.4.3 Children

The regression model for children accounted for 43% variance in the number of types of FA‐related psychological distress. The strongest variable was a greater number of symptoms (*β* = 0.35, 95% CI 0.18 to 0.24). Other variables that significantly contributed were living longer with FA, being older, a greater number of allergens, and country of residence (Table 6).

**TABLE 6:** Hierarchical regression model of variables associated with the types of FA related psychological distress in children (*n* = 1218).

Predictor	*N*	Model 1	Model 2
		Standardised | Unstandardised ***β* (95% CI)**	
Sum of allergens		0.08 | 0.07 (0.02 to 0.11)**	0.07 | 0.06 (0.02 to 0.10)**
Time living with food allergy		0.28 | 0.22 (0.16 to 0.27)***	0.25 | 0.19 (0.14 to 0.25)***
Sum of symptoms		0.35 | 0.21 (0.18 to 0.25)***	0.35 | 0.21 (0.18 to 0.24)***
Child's age		0.08 | 0.05 (0.01 to 0.10)*	0.11 | 0.07 (0.02 to 0.11)**
Sum of comorbidities		−0.00 | −0.01 (−0.15 to 0.13)	0.01 | 0.03 (−0.12 to 0.17)
Child prescribed an AAI			
No (reference)	455	–	–
Yes	715	0.07 | 0.48 (0.11 to 0.86)**	0.05 | 0.32 (−0.07 to 0.71)
Unsure	48	−0.04 | −0.68 (−1.45 to 0.09)	−0.05 | −0.75 (−1.52 to 0.03)
Child experienced anaphylaxis			
No (reference)	517	–	
Yes	617	0.05 | 0.32 (−0.07 to 0.70)	0.05 | 0.33 (−0.06 to 0.71)
Unsure	84	−0.02 | −0.23 (−0.82 to 0.37)	−0.02 | −0.25 (−0.84 to 0.33)
Child treated with adrenaline			
No (reference)	683		
Yes	485	0.01 | 0.07 (−0.31 to 0.44)	0.02 | 0.11 (−0.27 to 0.49)
Unsure	84	−0.02 | −0.33 (−1.08 to 0.42)	−0.02 | −0.26 (−1.01 to 0.48)
Country			
Canada (reference)	181	–	–
Australia	118	–	−0.01 | −0.10 (−0.69 to 0.50)
Brazil	182	–	−0.07 | −0.64 (−1.20 to −0.08)*
France	108	–	−0.07 | −0.84 (−1.45 to −0.23)**
Germany	101	–	−0.05 | −0.56 (−1.20 to 0.09)
Portugal	161	–	−0.10 | −0.91 (−1.45 to −0.38)***
Spain	143	–	−0.14 | −1.45 (−2.00 to −0.89)***
UK	110	–	−0.05 | −0.56 (−1.15 to 0.04)
USA	114	–	−0.08 | −0.86 (−1.45 to −0.26)**
*R* ^2^		0.416	0.432
Adjusted *R* ^2^		0.411	0.423
*R* ^2^ change		0.416	0.016
*F* statistic		78.23 (*p* < 0.001)	48.00 (*p* < 0.001)

*Note*: Due to insufficient participant numbers, Italy was excluded as a comparative group.

*p < 0.050, **p < 0.010, ***p < 0.001.

## 4 DISCUSSION

This is the first study to examine variables associated with specific types of FA‐related psychological distress in adults, caregivers and children across multiple countries. Hierarchical regression models incorporating clinical characteristics and country of residence explained between 43% and 54% variance in types of FA‐relatedpsychological distress in adults, caregivers, and children.

Whilst variables associated with distress varied across adults, caregivers and children, those that remained consistent were a higher number of symptoms and allergens. A higher number of symptoms may well be indicative of a higher number of past reactions or more severe reactions. In addition, a higher number of allergens may increase the risk of exposure and compound the implications of allergen avoidance.^21^

Whilst the psychological benefit or burden of carrying AAI prescription has shown to vary and sometimes conflict,^6^, ^13^, ^14^, ^15^ here AAI prescription increased the likelihood of adults and caregivers (and children—although this became non‐significant when country of residence was added) experiencing more types of distress. A possible explanation is that a prescription increases the burden of responsibility,^22^ represents a proxy measure for the individual having a more severe allergy^23^ and/or serves as a constant reminder of the fatality risk associated with living with an FA and thereby increases distress in various domains.

In adults and caregivers, an experience of anaphylaxis increased the likelihood of experiencing more types of distress, providing further support to existing research.^6^, ^9^, ^11^, ^24^ A review of parent experiences of anaphylaxis reported that not only did parents report fear, anxiety and panic but many parents had difficulty distinguishing between serious and less serious allergic symptoms.^25^ This could explain why adults who were unsure about the experience of anaphylaxis also experienced more distress because of fear and uncertainty about their level of risk.

For caregivers, children receiving treatment with AAI were associated with a smaller number of types of distress (although this became non‐significant when country of residence was added). It is possible that successfully managing a reaction to food increases self‐efficacy and lowers their perception of the risks associated with future reactions. Partial support for this comes from a study employing food challenges in a controlled setting, where 68 8–16‐year‐olds participated, 29% of which experienced anaphylaxis and self‐administered AAI. Significant improvements in FAQoL and self‐efficacy scores were observed in the overall cohort and caregiver FAQoL scores also improved.^26^

In adults, the presence of one or more FA‐related comorbid conditions contributed to explaining variance in types of distress, providing further support to existing studies that reported the presence of allergic diseases such as atopic dermatitis, asthma^5^, ^27^ or eosinophilic oesophagitis^6^ as an important predictor of FAQoL alongside FA.

The contrast between caregivers and children in relation to age and types of distress could be explained by the burden of responsibility. Parents of younger children report worse FAQoL^28^ and are responsible for avoiding allergens and carrying and administering medication.^29^ At around 8 years of age, due to an increase in cognitive abilities, children develop a greater awareness of the risk of FA and the possible consequences.^30^ They may start to take on more responsibility for the management of their FA as they experience social events away from the family sphere^29^, ^31^ and this is reflected in FAQoL scores: compared to 0–3 and 4–6 year olds, those aged 7–12 had a lower FAQoL.^10^ An unanticipated result in the present study was the increase in time a caregiver had lived with their child's FA associated with an increase in the types of distress reported. There is no research that focuses on time living with FA, but it may be that caregivers whose child had been living with FA for a longer time had had to manage FA in a greater number of settings, possibly experienced more episodes of anaphylaxis, and so reported a higher number of types of distress.

Being female was significantly associated with experiencing more types of distress among adults and caregivers, but not among children. This finding aligns with the results of other studies.^8^, ^32^ Whilst it is unclear what contributes to this difference, in caregivers this could be attributed to women predominantly undertaking caregiving burdens. Previous research has found that the more involved a parent is in the management and care of a child with FA, the more impacted they are by it.^33^ In adults, further investigation into why females report a wider variety of distress is needed.

Significant differences in education levels in adults and caregivers, and the general trend of those with a PhD or MD reporting fewer types of distress, could be related to health literacy and risk perceptions or access to more resources for allergy care or management. A note of caution is due here given the limited number of participants in the categories that showed significant differences compared to the rest of the sample. Existing research shows that those most highly educated (Masters, PhD or professional degree) are significantly less likely to rate the risks of FA as high^34^ and parents with a university education have a lower perception of anaphylaxis risk.^27^ Higher education levels are significantly related to increased health literacy.^35^ Those with higher health literacy may have better access to support for managing FA, leading to greater control and perceived risk reduction or more accurate risk perceptions. For example, the risk of fatal anaphylaxis is less than 1% of the total mortality risk,^36^ and FA‐related anaphylaxis incidence is lower than accidental death in the general European population.^37^

Variance in types of distress across countries may be explained by factors not measured in this study. Participants from the UK, Australia, Canada, and Brazil consistently reported more types of distress than participants from other countries. Possible explanations include differences in healthcare systems, culture, and society, and risk perceptions fuelled by media coverage. For example, increased exposure to media coverage of FA‐related fatalities is associated with increased anxiety in children with FA and their parents.^38^ In the UK, increasing numbers of headlines reporting FA fatalities imply an increased risk,^39^ whereas between 1998 and 2018, the death rate for anaphylaxis to food halved, while hospital admissions tripled.^40^ Differences may also have occurred due to variations in food allergen regulations and risk management, which can affect consumer trust and confidence in the ability to choose safe foods. Cost of alternative foods, limitations on leisure activities and other life events and the need for advocacy may also have varying impacts on psychological distress across countries.

### 4.1 Limitations

Limitations of this study include the categorical nature of much of the data related to types of distress, the lack of a validated distress scale that covers all of the types of distress those managing FA report, and the lack of data on participants' perception of the severity of their or their children's FA. Our planned follow‐up surveys will address these shortcomings by allowing participants to grade the frequency of experiencing each type of distress and perceived FA severity, and include validated FA QoL scales. Most participants identified as white, female or university educated, which is not representative of the demographics for most included countries, thus limiting the generalisability of findings. Due to the method of recruitment and the requirement to have internet access to participate, only those who saw the advert from patient organisations or social media or were part of the survey panel were able to participate.

## 5 CONCLUSION

To the best of our knowledge, this is the first global study to compare variables associated with the types of FA‐related psychological distress. Across adults, children, and caregivers, a higher number of symptoms and food allergies—and in adults and caregivers, AAI prescription—are associated with more types of distress, suggesting a group particularly in need of psychological intervention. Such intervention could entail educating patients on the realistic risks and consequences of living with FA, providing FA educational resources, and increasing self‐efficacy relating to the management of their or their child's FA, such as increasing confidence in successfully managing a reaction needing an AAI. Adults may find it beneficial to learn how to recognise the symptoms of anaphylaxis, enabling them to more accurately assess their level of risk. In families with an allergic child, caregivers require more psychological support when the child is younger. As the child ages, caregiver support can be tapered off as the child takes charge of their own FA management. The findings herein will be incorporated into the development of interventions to support adults and caregivers in living with their or their child's FA in Phase Three of the GAPS study.

## AUTHOR CONTRIBUTIONS

**S. E. M. Purser**: Writing—original draft; methodology; writing—review and editing; formal analysis. **C. J. Jones**: Conceptualization; investigation; methodology; writing—review and editing; funding acquisition. **J. L. P. Protudjer**: Conceptualization; investigation; methodology; writing—review and editing; funding acquisition. **L. J. Herbert**: Conceptualization; investigation; methodology; writing—review and editing; funding acquisition. **C. Screti**: Conceptualization; investigation; methodology; writing—review and editing. **C. Roleston**: Investigation; methodology; writing—review and editing. **E. Mattacola**: Investigation; methodology; writing—review and editing. **H. A. Brough**: Conceptualization; investigation; methodology; writing—review and editing; funding acquisition. **C. Warren**: Conceptualization; investigation; methodology; writing—review and editing. **L. Polloni**: Investigation; methodology; writing—review and editing. **A. F. Santos**: Investigation; methodology; writing—review and editing. **R. Gupta**: Investigation; methodology; writing—review and editing; **M. J. Marchisotto**: Conceptualization; investigation; funding acquisition; methodology; writing—review and editing. **R. C. Knibb**: Conceptualization; investigation; funding acquisition; writing—original draft; methodology; writing—review and editing; formal analysis; supervision; project administration; data curation.

## CONFLICT OF INTEREST STATEMENT

RCK: research funding from the National Institute for Health Research, Aimmune, National Peanut Board, Novartis and the Food Standards Agency and honoraria from Nutricia, Viatris, Stallergenes Greer and DBV Technologies. RCK is also Chair of the British Society for Allergy and Clinical Immunology Psychology Special Interest Group. JP: Section Head for Allied Health and Co‐Lead, Research Pillar, for the Canadian Society of Allergy and Clinical Immunology; sits on the steering committee for Canada's National Food Allergy Action Plan and reports consultancy for Ajinomoto Cambrooke, Nutricia, Novartis and ALK‐Abelló. HB: research funding from the NIH (NAIAD) and speaker honoraria from DBV Technologies. MJM: sits on the National Peanut Board Food Allergy Advisory Council and reports consultancy for Novartis. RG: receives research support from the National Institutes of Health (NIH) (R21 ID # AI135705, R01 ID # AI130348, U01 ID # AI138907), Food Allergy Research & Education (FARE), Melchiorre Family Foundation, Sunshine Charitable Foundation, the Walder Foundation, UnitedHealth Group, Thermo Fisher Scientific, Novartis, and Genentech. She serves as a medical consultant/advisor for Genentech, Novartis, Aimmune LLC, Allergenis LLC, and Food Allergy Research & Education (FARE). Dr. Gupta has ownership interest in Yobee Care Inc. CJJ: research funding from the National Institute for Health Research and the Food Standards Agency and honoraria from the National Institute for Health Research, Nutricia, Mead Johnson and Allergy UK. A.F. Santos reports grants from Medical Research Council (MR/M008517/1; MC/PC/18052; MR/T032081/1), Food Allergy Research and Education (FARE), the Immune Tolerance Network/National Institute of Allergy and Infectious Diseases (NIAID, NIH), Asthma UK (AUK‐BC‐2015‐01), BBSRC, Rosetrees Trust and the NIHR through the Biomedical Research Centre (BRC) award to Guy's and St Thomas' NHS Foundation Trust, during the conduct of the study; personal fees from Thermo Fisher Scientific, Novartis, Allergy Therapeutics, Stallergenes Greer, IgGenix, as well as research support from Thermo Fisher Scientific and IgGenix through a collaboration agreement with King's College London.

## Supporting information

Supporting Information S1

## Data Availability

The data that support the findings of this study are available on request from the corresponding author. The data are not publicly available due to privacy or ethical restrictions.
