# Supplementary material for: Associations with food allergy‐related psychological distress in a global sample of adults, children and caregivers
Source: Clin Transl Allergy. 2025 Jun 6;15(6):e70071. doi: 10.1002/clt2.70071 (PMC12143967; doi:10.1002/clt2.70071)
Supplement: Supplementary file 1 — Supporting Information S1 [file CLT2-15-e70071-s001.docx]

Table a) Characteristics of the survey participants (Adults N=1329; Caregivers N=1373).

| **Participant Characteristics** | **Adults**  **N (%)** | **Caregivers**  **N (%)** |
| --- | --- | --- |
| Country of residence |  |  |
| Australia | 120 (9.0%) | 118 (8.6%) |
| Brazil | 90 (6.8%) | 188 (13.7%) |
| Canada | 208 (15.7%) | 186 (13.5%) |
| France | 111 (8.4%) | 120 (8.7%) |
| Germany | 115 (8.7%) | 110 (8.0%) |
| Italy | 126 (9.5%) | 18 (1.3%) |
| Portugal | 130 (9.8%) | 171 (12.5%) |
| Spain | 145 (10.9%) | 151 (11%) |
| United Kingdom | 116 (8.7%) | 118 (8.6%) |
| United States of America | 122 (9.2%) | 121 (8.8%) |
| Other | 46 (3.5%) | 72 (5.3%) |
| Age (years) (mean) | 38.1 (SD=13.4) | 37.6 (SD=8.8) |
| Sex (% Female) | 931 (70.2%) | 1031 (75.1%) |
| Ethnicity |  |  |
| Asian | 25 (1.9%) | 40 (2.9%) |
| Black, Black British, Caribbean, or African | 23 (1.7%) | 24 (1.7%) |
| Mixed or multiple ethic groups | 82 (6.2%) | 103 (7.5%) |
| White | 1135 (85.4%) | 1118 (81.4%) |
| Other ethnic group | 51 (3.8%) | 56 (4.1%) |
| Unknown | 2 (0.2%) | 3 (0.2%) |
| Prefer not to say | 11 (0.8%) | 29 (2.1%) |
| Highest level of education |  |  |
| No qualifications | 4 (0.3%) | 26 (1.9%) |
| School level | 212 (16.1%) | 140 (10.2%) |
| College level | 359 (27.2%) | 384 (28.0%) |
| University undergraduate level | 382 (29.0%) | 409 (29.8%) |
| University postgraduate level | 259 (19.7%) | 304 (22.1%) |
| PhD or MD | 54 (4.1%) | 74 (5.4%) |
| Other | 48 (3.6%) | 32 (2.3%) |
| Employment status |  |  |
| Full-time employment | 714 (55.0%) | 790 (57.5%) |
| Part-time employment | 198 (15.3%) | 272 (19.8%) |
| Not employed outside the home | 208 (16.0%) | 138 (10.1%) |
| Student | 146 (11.2%) | 42 (3.1%) |
| Marital status (% Married) | 558 (42.7%) | 934 (68.0%) |

Table b) Food allergy characteristics of adults and children (Adults N=1329; Caregivers N=1373).

| **Food Allergy Characteristics of adults and children** | **Adults**  **N (%)** | **Children**  **N (%)** |
| --- | --- | --- |
| Age (years) (mean) | 38.1 (SD=13.4) | 8.1 (SD=5.3) |
| Age of food allergy diagnosis (years) (mean) | 18.0 (SD=13.6) | 3.1  (SD=3.7) |
| Time living with food allergy (years) (mean) | 20.0 (SD=13.5) | 5.1  (SD=4.2) |
|  |  |  |
| Allergens* |  |  |
| Peanut | 480 (36.1%) | 571 (41.6%) |
| Almonds | 269 (20.2%) | 238 (17.3%) |
| Hazelnuts | 303 (22.8%) | 291 (21.2%) |
| Brazil nuts | 224 (16.9%) | 216 (15.7%) |
| Walnuts | 300 (22.6%) | 298 (21.7%) |
| Cashew nuts | 247 (18.6%) | 307 (22.4%) |
| Pecans | 218 (16.4%) | 204 (14.9%) |
| Pistachios | 236 (17.8%) | 276 (20.1%) |
| Macadamia nuts | 209 (15.7%) | 190 (13.8%) |
| Cow’s milk | 357 (26.9%) | 618 (45.0%) |
| Egg | 171 (12.9%) | 472 (34.4%) |
| Cereals containing gluten | 184 (13.8%) | 169 (12.3%) |
| Wheat | 152 (11.4%) | 128 (9.3%) |
| Soy | 146 (11.0%) | 170 (12.4%) |
| Crustaceans | 246 (18.5%) | 142 (10.3%) |
| Molluscs | 161 (12.1%) | 85 (6.2%) |
| Fish | 147 (11.1%) | 123 (9.0%) |
| Sesame | 84 (6.3%) | 117 (8.5%) |
| Celery | 39 (2.9%) | 28 (2.0%) |
| Lupin | 44 (3.3%) | 29 (2.1%) |
| Mustard | 57 (4.3%) | 47 (3.4%) |
| Sulphites and sulphur dioxide | 78 (5.9%) | 25 (1.8%) |
| Other | 429 (32.3%) | 285 (20.8%) |
| Food allergy diagnosis method* |  |  |
| Skin test | 698 (52.5%) | 785 (57.2%) |
| Blood test | 575 (43.3%) | 764 (55.6%) |
| Oral food challenge | 212 (16%) | 336 (24.5%) |
| History | 544 (40.9%) | 628 (45.7%) |
| Elimination diet | 220 (16.6%) | 251 (18.3%) |
| Other | 89 (6.7%) | 44 (3.2%) |
| Do not remember/unsure | 59 (4.4%) | 18 (1.3%) |
| Comorbid conditions* |  |  |
| Lactose intolerance | 409 (30.8%) | 291 (21.2%) |
| Eosinophilic esophagitis | 58 (4.4%) | 76 (5.5%) |
| Eosinophilic gastritis or other gastrointestinal disorder | 130 (9.8%) | 104 (7.6%) |
| Celiac disease | 109 (8.2%) | 75 (5.5%) |
| Eczema | 384 (28.9%) | 496 (36.1%) |
| Asthma | 442 (33.3%) | 422 (30.7%) |
| Environmental allergies | 486 (36.6%) | 422 (30.7%) |
| Oral allergy syndrome/pollen food syndrome | 175 (13.2%) | 96 (7.0%) |
| Drug allergy | 309 (23.3%) | 101 (7.4%) |
| None of the above | 165 (12.4%) | 263 (19.2%) |
| Adrenaline auto-injector prescription | 652 (49.1%) | 787 (57.3%) |
| History of anaphylaxis | 666 (50.1%) | 691 (50.3%) |
| * The items listed under these headings are not mutually exclusive, i.e. participants may fall into multiple categories | | |

The sum of 34 symptoms participants could report as a reaction to food included: Cough/sneezing; Runny nose; Irritable/itchy nose; Congested nose; Wheezing; Tight chest; Breathless; Asthma; Rash, wheal or ‘welts’ or ‘nettle rash’ on skin; Itchy skin; Dry skin; Scabby skin; Swelling of face, eyes, lips; Swelling of tongue/roof of the mouth; Worsening eczema; Bloated stomach; Abdominal pain/stomach cramps; Heart burn; Sickness/vomiting; Diarrhoea; Blood in stool; Dehydration; Loss of weight/malnutrition; Excessive wind; Irritable bowel type symptoms; Tingling in mouth, throat or ear; Itching in mouth, throat or ear; Throat tightness/swelling; Tongue swelling; Anaphylaxis; Collapse/faint/loss of consciousness; Seizure; Incontinence; Chest pain

Table c) Education levels and types of distress

|  | **Adults** | | **Caregivers** | |
| --- | --- | --- | --- | --- |
| **Education Level** | **N** | **Mean number of types of distress experienced (SD)** | **N** | **Mean number of types of distress experienced (SD)** |
| School level qualifications | 212 (16.1%) | 5.02 (5.51) | 140  (10.2%) | 6.29 (6.52) |
| College level qualifications | 359 (27.2%) | 4.74 (5.17) | 384  (28,0%) | 6.99 (6.12) |
| University undergraduate level qualifications | 382 (29%) | 5.46 (5.56) | 409  (29.8%) | 7.35 (6.15) |
| University postgraduate level qualifications | 259 (19.7%) | 5.29 (5.02) | 304  (22.1%) | 7.04 (5.91) |
| PhD or MD | 54  (4.1%) | 3.11 (3.80) | 74  (5.4%) | 5.53 (5.30) |
| No qualifications | 4  (0.3%) | 3.00 (2.94) | 26  (1.9%) | 11.08 (5.60) |
| Other qualifications | 48  (3.6%) | 6.50 (5.57) | 32  (2.3%) | 10.94 (5.91) |

Table d) Country differences in adult and caregiver types of distress

|  | **Country (N, %)** | | | | | | | | | |  |  | |  | |  | |  | | |
| --- | --- | --- | --- | --- | --- | --- | --- | --- | --- | --- | --- | --- | --- | --- | --- | --- | --- | --- | --- | --- |
| **Type of FA-related psychological distress** | **Australia** | | **Brazil** | | **Canada** | | **France** | | **Germany** | | **Italy** | **Portugal** | | **Spain** | | **UK** | | **USA** | |  |
|  | Adults (n=120) | Care-givers  (n=118) | Adults  (n=90) | Care-givers  (n=188) | Adults (n=208) | Care-givers  (n=186) | Adults (n=111) | Care-givers  (n=120) | Adults (n=115) | Care-givers  (n=110) | Adults (n=126) | Adults (n=130) | Care-givers (n=171) | Adults (n=145) | Care-givers  (n=151) | Adults (n=116) | Care-givers  n=118) | Adults (n=122) | Care-givers  (n=121) |  |
| Anxiety about an allergic reaction / Anxiety about my child having an allergic reaction | 77 (64.2%) | 107 (90.7%) | 68 (75.6%) | 134  (71.3%) | 141 (67.8%) | 122  (65.6%) | 24 (21.6%) | 31  (25.8%) | 33 (28.7%) | 33  (30%) | 50  (39.7%) | 42  (32.3%) | 89  (52%) | 53  (36.6%) | 50  (33.1%) | 91  (78.4%) | 76  (64.4%) | 46  (37.7%) | 69  (57%) |  |
| Anxiety about living with my FA / Anxiety about living with my child's food allergy | 70 (58.3%) | 92  (78%) | 64 (71.1%) | 151  (80.3%) | 123 (59.1%) | 104  (55.9%) | 35 (31.5%) | 36  (30%) | 29  (25.2%) | 34  (30.9%) | 54  (42.9%) | 31  (23.8%) | 76  (44.4%) | 51  (35.2%) | 40  (26.5%) | 82  (70.7%) | 74  (63.2%) | 47  (38.5%) | 71  (58.7%) |  |
| Worry about getting allergen free/safe foods / Worry or anxiety about getting allergen free/safe foods for my child | 53 (44.2%) | 84  (71.2%) | 63  (70%) | 149  (79.3%) | 105 (50.5%) | 79  (42.5%) | 9  (8.1%) | 4  (3.3%) | 11  (9.6%) | 10  (9.1%) | 36  (28.6%) | 21  (16.2%) | 62  (36.3%) | 33  (22.8%) | 28  (18.5%) | 70  (60.3%) | 46  (39%) | 20  (16.4%) | 38  (31.4%) |  |
| Sadness about the impact of FA on my life / Sadness about the impact of food allergy on my/my child’s life | 40 (33.3%) | 98 (83.1%) | 63  (70%) | 131  (69.7%) | 100  (48.1%) | 92  (49.5%) | 13  (11.7%) | 10  (8.3%) | 21 (18.3%) | 14  (12.7%) | 29  (23%) | 26  (20%) | 56  (32.7%) | 36  (24.8%) | 31  (20.5%) | 64  (55.2%) | 60  (50.8%) | 23  (18.9%) | 46  (38%) |  |
| Worry of a potentially fatal reaction because of my FA / Worry of my child experiencing a potentially fatal reaction | 49 (40.8%) | 97  (82.2%) | 41 (45.6%) | 100  (53.2%) | 103 (49.5%) | 38  (20.4%) | 5  (4.5%) | 9  (7.5%) | 5  (4.3%) | 12  (10.9%) | 26  (20.6%) | 15  (11.5%) | 65  (38%) | 29  (20%) | 34  (22.5%) | 79  (68.1%) | 65  (55.1%) | 19  (15.6%) | 44  (36.4%) |  |
| Worry about not being able to take part in social activities because of my FA / Worry about not being able to take part in social activities because of my child's food allergy | 48  (40%) | 76  (64.4%) | 51 (56.7%) | 103  (54.8%) | 90 (43.3%) | 69  (34.9%) | 7  (6.3%) | 11  (9.2%) | 5  (4.3%) | 9  (8.2%) | 21  (16.7%) | 11  (8.5%) | 37  (21.6%) | 27  (18.6%) | 26  (17.2%) | 65  (56%) | 52  (44.1%) | 13  (10.7%) | 35  (28.9%) |  |
| Stress of managing my health because of my FA / Stress of managing my child's health | 39 (32.5%) | 74  (62.7%) | 44 (48.9%) | 92  (48.9%) | 70  (33.7%) | 64  (34.4%) | 7  (6.3%) | 13  (10.8%) | 10 (8.7%) | 13  (11.8%) | 28  (22.2%) | 16  (12.3%) | 41  (24%) | 29  (20%) | 23  (15.2%) | 42  (36.2) | 42  (35.6%) | 23  (18.9%) | 42  (34.7%) |  |
| Worry about telling people I have a FA / Worry about telling people my child has a food allergy | 38  (31.7) | 40  (33.9%) | 43 (47.8%) | 77  (41%) | 69 (33.2%) | 75  (40.3%) | 10  (9%) | 5  (4.2%) | 6  (5.2%) | 3  (2.7%) | 24  (19%) | 11  (8.5%) | 25  (14.6%) | 17  (11.7%) | 9  (6%) | 59  (50.9%) | 23  (19.5%) | 10  (8.2%) | 16  (13.2%) |  |
| Anxiety about having an unnecessarily restricted diet / Fear of restricting my child's diet | 34 (28.3%) | 55  (46.6%) | 38 (42.2%) | 79  (42%) | 59 (28.4%) | 51  (27.4%) | 4  (3.6%) | 11  (9.2%) | 20  (17.4%) | 21  (19.1%) | 17 (13.5%) | 12  (9.2%) | 19  (11.1%) | 25  (17.2%) | 16  (10.6%) | 41  (35.3%) | 32  (27.1%) | 23  (18.9%) | 33  (27.3%) |  |
| Anxiety about administering my AAI / Anxiety about administering adrenaline/ epinephrine to my child | 29 (24.2%) | 50  (42.4%) | 12 (13.3%) | 37  (19.7%) | 71 (34.1%) | 70  (37.6%) | 6  (5.4%) | 9  (7.5%) | 8  (7%) | 12  (10.9%) | 21  (16.7%) | 3  (2.3%) | 35  (20.5%) | 15  (10.3%) | 23  (15.2%) | 47  (40.5%) | 39  (33.1%) | 17  (13.9%) | 34  (28.1%) |  |
| Worry about carrying my AAI / Worry about having to carry my child’s adrenaline /epinephrine | 30  (25%) | 52  (44.1%) | 10  (11.1%) | 46  (24.5%) | 53 (25.5%) | 45  (24.2%) | 1  (0.9%) | 4  (3.3%) | 4  (3.5%) | 5  (4.5%) | 18  (14.3%) | 10  (7.7%) | 23  (13.5%) | 19  (13.1%) | 19  (12.6%) | 49  (42.2%) | 34  (28.8%) | 14  (11.5%) | 25  (20.7%) |  |
| FA-related bullying / My child experiencing food allergy-related bullying | 24  (20%) | 50 (42.4%) | 40 (44.4%) | 50  (26.6%) | 50  (24%) | 66  (35.5%) | 7  (6.3%) | 3  (2.5%) | 3  (2.6%) | 7  (6.4%) | 6  (4.8%) | 5  (3.8%) | 19  (11.1%) | 7  (4.8%) | 8  (5.3%) | 37  (31.9%) | 40  (33.9%) | 10  (8.2%) | 38  (31.4%) |  |
| Panic attacks because of my FA/ Panic attacks because of my child's food allergy | 37 (30.8%) | 26  (22%) | 11  (12.2%) | 36  (19.1%) | 48  (23.1%) | 36  (19.4%) | 5  (4.5%) | 8  (6.7%) | 4  (3.5%) | 8  (7.3%) | 12  (9.5%) | 12  (9.2%) | 10  (5.8%) | 10  (6.9%) | 3  (2%) | 33  (28.4%) | 15  (12.7%) | 13  (10.7%) | 19  (15.7%) |  |
| Anxiety about oral food challenges / Anxiety about my child experiencing oral food challenges | 16 (13.3%) | 48  (40.7%) | 8  (8.9%) | 40  (21.3%) | 46 (22.1%) | 56  (30.1%) | 7  (6.3%) | 13  (10.8%) | 8  (7%) | 16  (14.5%) | 17  (13.5%) | 5  (3.8%) | 32  (18.7%) | 64  (11%) | 14  (9.3%) | 13  (11.2%) | 30  (25.4%) | 29  (23.8%) | 49  (40.5%) |  |
| Worry about access to AAIs / Worry or anxiety about access to adrenaline/epinephrine auto-injectors | 26 (21.7%) | 43  (36.4%) | 11  (12.2%) | 44  (23.4%) | 36 (17.3%) | 38  (20.4%) | 3  (2.7%) | 3  (2.5%) | 4  (3.5%) | 6  (5.5%) | 13  (10.3%) | 6  (4.6%) | 16  (9.4%) | 11  (7.6%) | 14  (9.3%) | 36  (31%) | 46  (39%) | 3  (2.5%) | 23  (19%) |  |
| Worry about finding an intimate partner because of my FA | 18  (15%) |  | 18  (20%) |  | 37  (17.8%) |  | 6  (5.4%) |  | 2  (1.7%) |  | 12  (9.5%) | 5  (3.8%) |  | 12  (8.3%) |  | 28  (24.1%) |  | 8  (6.6%) |  |  |
| Worry about finding or keeping a safe job because of my FA | 24  (20%) |  | 23 (25.6%) |  | 44 (21.2%) |  | 0  (0%) |  | 2  (1.7%) |  | 6  (4.8%) | 5  (3.8%) |  | 8  (5.5%) |  | 26  (22.4%) |  | 6  (4.9%) |  |  |
| Needle phobia or other medical procedure anxiety | 11  (9.2%) |  | 10  (11.1%) |  | 17  (8.2%) |  | 2  (1.8%) |  | 7  (6.1%) |  | 7  (5.6%) | 6  (4.6%) |  | 7  (4.8%) |  | 11  (9.5%) |  | 2  (1.6%) |  |  |
| Anxiety about avoiding unsafe foods | 68 (56.7%) |  | 67 (74.4%) |  | 120 (57.7%) |  | 22 (19.8%) |  | 21  (18.3%) |  | 45  (35.7%) | 30  (23.1%) |  | 48  (33.1%) |  | 78  (67.2%) |  | 32  (26.2%) |  |  |
| Loss of a normal life due to my FA | 40 (33.3%) |  | 56 (62.2%) |  | 83  (39.9%) |  | 15 (13.5%) |  | 19  (16.5%) |  | 22  (17.5%) | 21  (16.2%) |  | 31  (21.4%) |  | 53  (45.7%) |  | 21  (17.2%) |  |  |
| Fear of trusting others to know what to do if I have an allergic reaction | 46 (38.3%) |  | 44 (48.9%) |  | 91 (43.8%) |  | 13 (11.7%) |  | 7  (6.1%) |  | 24  (19%) | 17  (13.1%) |  | 25  (17.2%) |  | 70  (60.3%) |  | 19  (15.6%) |  |  |
| Fear for my child’s safety |  | 95  (80.5%) |  | 139  (73.9% |  | 105  (56.5%) |  | 30  (25%) |  | 22  (20%) |  |  | 93  (54.4%) |  | 47  (31.1%) |  | 76  (64.4%) |  | 65  (53.7%) |  |
| Fear of trusting others with care of my child |  | 110  (93.2%) |  | 164  (87.2%) |  | 112  (60.2%) |  | 18  (15%) |  | 19  (17.3%) |  |  | 86  (50.3%) |  | 42  (27.8%) |  | 72  (61%) |  | 57  (47.1%) |  |
| Worry about cost |  | 31  (26.3%) |  | 104  (55.3%) |  | 30  (16.1%) |  | 3  (2.5%) |  | 12  (10.9%) |  |  | 26  (15.2%) |  | 8  (5.3%) |  | 9  (7.6%) |  | 16  (13.2%) |  |
| Worry after my child has a severe reaction |  | 76  (64.4%) |  | 108  (57.4%) |  | 92  (49.5%) |  | 12  (10%) |  | 9  (8.2%) |  |  | 49  (28.7%) |  | 32  (21.2%) |  | 48  (40.7%) |  | 41  (33.9%) |  |
| Worry that people won’t understand the seriousness of my child’s allergies even though I try to explain it to them |  | 72  (61%) |  | 114  (60.6%) |  | 58  (31.2 %) |  | 2  (1.7%) |  | 8  (7.3%) |  |  | 37  (21.6%) |  | 22  (14.6%) |  | 46  (39%) |  | 40  (33.1%) |  |
| Social isolation during routine activities due to my child's food allergy |  | 102  (86.4%) |  | 153  (81.4%) |  | 97  (52.2%) |  | 5  (4.2%) |  | 7  (6.4%) |  |  | 76  (44.4%) |  | 35  (23.2%) |  | 65  (55.1%) |  | 47  (38.8%) |  |
| Cells with an orange fill shows where the adjusted residual was ≤2, which shows that significantly more participants reported that type of distress than expected. Cells with a green fill shows cells where the adjusted residual was ≤-2 which shows that significantly fewer participants than expected reported distress.  Due to insufficient participant numbers, Italy was excluded as a comparative group for caregivers. | | | | | | | | | | | | | | | | | | | |  |

Table e) Country differences in child types of distress as reported by their caregiver

|  | **Country (N, %)** | | | | | | | | |
| --- | --- | --- | --- | --- | --- | --- | --- | --- | --- |
| **Type of FA-related psychological distress** | *Australia*  *(n=118)* | *Brazil (n=188)* | *Canada (n=186)* | *France (n=120)* | *Germany*  *(n=110)* | *Portugal*  *(n=171)* | *Spain*  *(n=151)* | *UK*  *(n=118)* | *USA*  *(n=121)* |
| *Anxiety about living with food allergy* | 50  (42.4%) | 51  (27.1%) | 68  (36.6%) | 37  (30.8%) | 23  (20.9%) | 41  (24%) | 26  (17.2%) | 51  (43.2%) | 52  (43 %) |
| *Anxiety about oral food challenges* | 39  (33.1%) | 18  (9.6%) | 39  (21%) | 13  (10.8%) | 11  (10%) | 21  (12.3%) | 14  (9.3%) | 32  (27.1%) | 31  (25.6%) |
| *Anxiety about an allergic reaction* | 62  (52.5%) | 47  (25%) | 80  (43%) | 19  (15.8%) | 26  (23.6%) | 39  (22.8%) | 16  (10.6%) | 54  (45.8%) | 42  (34.7%) |
| *Anxiety about administering adrenaline/epinephrine* | 40  (33.9%) | 12  (6.4%) | 57  (30.6%) | 11  (9.2%) | 7  (6.4%) | 22  (12.9%) | 13  (8.6%) | 28  (23.7%) | 29  (24%) |
| *Food allergy-related bullying* | 22  (18.6%) | 24  (12.8%) | 29  (15.6%) | 7  (5.8%) | 15  (13.6%) | 14  (8.2%) | 9  (6%) | 21  (17.8%) | 13  (10.7%) |
| *Panic attacks because of food allergy* | 11  (9.3%) | 8  (4.3%) | 21  (11.3%) | 9  (7.5%) | 13  (11.8%) | 5  (2.9%) | 7  (4.6%) | 13  (11%) | 17  (14%) |
| *Sadness about the impact of food allergy on his/her life* | 62  (52.5%) | 62  (33%) | 71  (38.2%) | 12  (10%) | 13  (11.8%) | 45  (26.3%) | 24  (15.9%) | 47  (39.8%) | 40  (33.1%) |
| *Social isolation during routine activities* | 50  (42.4%) | 50  (26.6%) | 42  (22.6%) | 11  (9.2%) | 11  (10%) | 17  (9.9%) | 19  (12.6%) | 33  (28%) | 32  (26.4%) |
| *Stress of managing health* | 19  (16.1%) | 17  (9%) | 24  (12.9%) | 11  (9.2%) | 9  (8.2%) | 8  (4.7%) | 12  (7.9%) | 16  (13.6%) | 14  (11.6%) |
| *Worry about having to carry adrenaline/epinephrine* | 28  (23.7%) | 9  (4.8%) | 39  (21%) | 2  (1.7%) | 2  (1.8%) | 16  (9.4%) | 9  (6%) | 22  (18.6%) | 13  (10.7%) |
| *Worry about not being able to take part in social activities because of food allergy* | 47  (39.8%) | 45  (23.9%) | 55  (29.6%) | 8  (6.7%) | 11  (10%) | 29  (17%) | 15  (9.9%) | 36  (30.5%) | 24  (19.8%) |
| *Worry about telling people about food allergy* | 39  (33.1%) | 29  (15.4%) | 42  (22.6%) | 10  (8.3%) | 5  (4.5%) | 20  (11.7%) | 7  (4.6%) | 30  (25.4%) | 25  (20.7%) |
| *Worry of a potentially fatal reaction* | 38  (32.2%) | 31  (16.5%) | 50  (26.9%) | 6  (5%) | 10  (9.1%) | 26  (15.2%) | 15  (9.9%) | 35  (29.7%) | 25  (20.7%) |

Cells with an orange fill shows where the adjusted residual was ≤2, which shows that significantly more participants reported that type of distress than expected. Cells with a green fill shows cells where the adjusted residual was ≤-2 which shows that significantly fewer participants than expected reported distress. Due to insufficient participant numbers, Italy was excluded as a comparative group.
